# Use of carbon-13 as a population marker for *Anopheles arabiensis *in a sterile insect technique (SIT) context

**DOI:** 10.1186/1475-2875-5-6

**Published:** 2006-01-30

**Authors:** Rebecca Hood-Nowotny, Leo Mayr, Bart GJ Knols

**Affiliations:** 1 International Atomic Energy Agency (IAEA), Agency's Laboratories Seibersdorf, A-2444 Seibersdorf, Austria; 2 Laboratory of Entomology, Wageningen University and Research Center, P.O. Box 8031, 6700 EH Wageningen, The Netherlands

## Abstract

**Background:**

Monitoring of sterile to wild insect ratios in field populations can be useful to follow the progress in genetic control programmes such as the Sterile Insect Technique (SIT). Of the numerous methods for marking insects most are not suitable for use in mass rearing and mass release. Suitable ones include dye marking, genetic marking and chemical marking.

**Methods:**

The feasibility of using the stable isotope of carbon, ^13^C, as a potential chemical marker for *Anopheles arabiensis *was evaluated in the laboratory. Labeled-^13^C glucose was incorporated into the larval diet in a powder or liquid form. The contribution of adult sugar feeding to the total mosquito carbon pool and the metabolically active carbon pool was determined by tracing the decline of the enrichment of the adult male mosquito as it switched from a labeled larval diet to an unlabeled adult diet. This decline in the adult was monitored by destructive sampling of the whole mosquito and analyzed using isotope ratio mass spectrometry.

**Results:**

A two-pool model was used to describe the decline of the ^13^C-enrichment of adult mosquitoes. The proportion of the total adult carbon pool derived from the adult sugar diet over the life span of mosquitoes was determined and the ratio of structural carbon, with a low turnover rate to metabolically active non-structural carbon was assessed. The uptake and turnover of sugar in the metabolically active fraction suggests that after 3 days >70% of the active fraction carbon is derived from sugar feeding (increasing to >90% by day 7), indicating the high resource demand of male mosquitoes.

**Conclusion:**

It was possible to "fix" the isotopic label in adult *An. arabiensis *and to detect the label at an appropriate concentration up to 21 days post-emergence. The optimum labeling treatment would cost around 250 US$ per million mosquitoes. Stable isotope marking may thus aid research on the fate of released insects besides other population-based ecological studies.

## Background

Monitoring of sterile to wild insect ratios can be useful to follow the progress in genetic control programmes integrating the Sterile Insect Technique (SIT). SIT campaigns are initiated with conventional pre-release population suppression methods and then followed by sequential mass release of factory-reared and sterilized males until the sterile-to-wild ratios exceed 10:1 [1]. Field sampling will yield both released and wild insects and pre-release marking is necessary to differentiate both groups. There are numerous methods for marking insects as reviewed by Hagler and Jackson [2]. Some of these are clearly not suitable for mass-rearing such as individual marking, numbering and coding. Methods which are suitable include dye or fluorescent powder marking [3-5] that can be observed directly or with an ultraviolet light source, genetic marking [4] or chemical marking [6-8]. Stable isotope labeling falls in this latter category.

Stable isotopes are naturally abundant in the environment, safe and non-radioactive [9]. It should be noted, therefore, that stable isotopes are very different from the radioactive isotopes used for insect marking until the 1970s [10]. An isotope of an element has the same atomic number but a different number of nuclei and thus a different atomic weight. Stable isotopes react chemically in the same way as the more common isotopes. Although different rates of reaction can result in slight variations in isotopic composition in nature, this discrimination is generally irrelevant in labeling experiments. Intrinsic variations in natural abundance of stable isotopes have been used extensively in ecological studies of insects to trace food-web structure, migration patterns, feeding preferences etc. [11]. However, few have used enriched labeling techniques for marking and subsequent ecological investigations [12,13].

It is possible to purchase substances highly enriched in the rarer stable isotopes that can be easily integrated into insect feeding regimes. Appropriate isotope feeding management ensures that the isotopic composition of the food source is fixed into structural body tissue of the target organism [9,12] It is subsequently possible to use the isotopic signature as a unique marker. Stable isotope marking of insects fulfills the marking criteria set out by Hagler and Jackson [2] of a) retention, b) not affecting the insect's fecundity or behavior, c) durable, d) non-toxic, e) easily applied, f) clearly identifiable and g) inexpensive. While the latter could be contested, recent advances in both mass spectrometry and stable isotope measurement using laser technologies have driven the cost of analysis down to levels now comparable to other chemical and molecular analyses [9]. In insect studies in particular, the costs of the enriched feeding substances are not prohibitively high, given the large numbers of insects that may be marked with minimal amounts of enriched material. For example Hershey *et al *[9,12] labeled a section of a river using ^15^N-fertilizer to study the mayfly drift paradox.

There is considerable interest in the role of sugar feeding in mosquitoes [14] and its role in survival and reproductive success [15-17]. Most studies have focused on sugar feeding of females and how this affects their vectorial capacity [18,19] with only limited interest in sugar feeding of males. However, as the sole source of food for males, and of significance in terms of the ability to engage in mating activities [20], this behaviour is vitally important for strategies in which males are released in the field with the aim to effectively compete with wild males for virgin females. Moreover, sugar feeding is routinely used for mosquito colony maintenance and the search for additional food sources that can be used in mass rearing is still in its infancy.

Nutrient allocation has been difficult to measure quantitatively and stable isotopes have proved to be a useful tool in studying the uptake and turnover of nectar in hawkmoths [21,22]. Considering the above, stable isotopes were used to study uptake and turnover of sugar feeding in the male mosquito *Anopheles arabiensis*, with the aim to develop a labeling technique for marking mosquitoes for ecological and population-based studies.

## Materials and methods

*An. arabiensis *mosquitoes (KGB strain; originally from Zimbabwe) were used in all experiments. 200 eggs were placed in plastic trays (20 × 20 cm), lined with 2 cm strips of cellulose filter paper and filled with 500 ml of deionized water. The trays were placed on heating mats set at 36°C between 14.00-10.00 39°C between 10.00–14.00, giving water temperatures of 27°C and 30°C, respectively. Light intensity in the insectary was approximately 2,000 lux during the day and was dimmed between 16.30–18.00 hrs till full darkness. A dawn period was simulated between 06.30–07.30 (from 0–2,000 Lux). Adults were kept in standard 30 × 30 × 30 mosquito rearing cages and maintained at a temperature of 27°C and relative humidity of 80%.

Larvae were fed daily with 0.05 g/tray of the either regular or labeled fish food (AquariCare Koi Floating Blend, USA) dependent on the treatment. Fish food was ground and sieved through a 224 μm sieve.

Labeled treatments (treatments T1-T3) were prepared by mixing five grams of dried ground fish food with solid labeled glucose, the mixtures were homogenized overnight in plastic vials containing steel rods and rotated using a small roller device.

In the glucose solution treatments (treatments T5-T7), ^13^C-labeled stock glucose solution was prepared in deionized-distilled water. This stock solution was added at the same time as the eggs were added to the trays. The trays were covered with mosquito netting.

Treatments T1-T3 contained 0.5, 2.5 or 5 mg of 99 atom % ^13^C-glucose per gram of fish food. Treatment 4 served as the control of unlabeled fish food only (T4). Treatments T5-T7 consisted of 0.175, 0.875 or 1.75 mg of 99 atom % ^13^C glucose added to trays as a solution besides regular fish food. Daily, 0.05 g of the appropriate fish food and 50 ml of deionized water was added to each tray until an appropriate number of adults emerged.

Approximately 100–150 adult mosquitoes were captured and counted into separate cages using a suction device. Custom-made mosquito cages consisted of 20 cm diameter, 20 cm high PVC piping, permanently sealed on one end with mosquito netting and PVC tape; at the other end a mosquito net sleeve was attached to allow access. Adult mosquitoes were fed *ad libitum *using sugar-feeding devices suspended in the cage containing 20 ml of unlabeled 10% w/v sugar solution. These devices consisted of 100 ml screw lid plastic containers with a hole cut out of the lids and sealed with 20 micron polyester mesh material. The feeding devices and cages were tested in pilot experiments and found to be suitable for the study (M. Benedict and H. Bossin, unpublished data).

Mosquitoes were sampled at pupae stage and 3, 7, 14, 21 or 28 days post emergence. Five replicate males per treatment were sampled, only 3 replicates were sampled at 21 and 28 days due to lack of material. Whole mosquitoes were put into tin cups 8 by 5 mm and dried overnight in an oven at 50°C. Subsequently, the cups were sealed and contents analyzed using a Carlo Erba (Milan, Italy) carbon nitrogen (CN) analyser, linked to an Optima isotope ratio mass spectrometer (IRMS) (GV Instruments, Manchester, UK). Briefly, prepared samples were combusted in the CN analyser in an atmosphere of oxygen at 1,800°C. Following complete oxidation, the sample was carried in a stream of helium through a series of scrubbers to remove sulphurous impurities and residual water, and over hot copper to reduce oxides of nitrogen to elemental nitrogen (N_2_). Carbon dioxide (CO_2_) and N_2 _peaks were separated out on a 3 m Porapak Q gas chromatography column. The CO_2 _peak was bled into the mass spectrometer.

### Sample analysis

The measurement of isotopic composition for a particular element is commonly based on the ratio of the less abundant isotope of interest to the more abundant isotope. In natural abundance or micro enrichment studies values are generally reported as ratios of the lighter to the heavier isotope referenced against international standards in delta (δ) units parts per thousand ‰. A lower-case delta value is defined as the isotopic ratio of a sample standardized to the isotopic ratio of a defined reference:

[(R_x _- R_s_) / R_S_] × 1000 = δ     (1)

Where R_x _is the isotopic ratio of the sample and R_s _is the isotopic ratio of the reference standard. The defined reference standard for carbon-13 has been Belemnite of the Pee Dee Formation in South Carolina, U.S. (Vienna PDB), with ^13^C/^12^C = 11237.2 ± 60 × 10^-6 ^[23].

The proportion of C derived from sugar feeding was determined using a simple two-source mixing model equation 2. A two pool mixing model assumes the final pool (with a known isotopic enrichment) is derived solely from two source pools of known isotopic enrichment. It is therefore possible to determine the percentage contribution of each source pool to the final pool using the mixing model equation. The measured ^13^C of the sugar (3 replicates) was 23.2‰ vs PDB (1.0858 atom %) and the enrichment of the pupae was taken as the average value. It should be noted that all values were converted to atom % prior to calculation to ensure validity of equations and avoid conflicting minuses.

% C derived from total adult sugar diet =

{1- [(F^13^C mosquito-F^13^C sugar)/(F^13^Cpupae-F^13^Csugar)]}*100     (2)

Where F = atom fraction which is equal to abundance/100

% C of active fraction derived from sugar diet =

{1- [(F^13^C mosquito-YF^13^C sugar-YF^13^Cpupae)/(F^13^Cpupae-F^13^Csugar)]}*100     (3)

Where Y = % C derived from pupae at leveling point on the curve which represents the structural fraction which is turning over slowly (see results and discussion) for further description. This was calculated to be between 49 and 50% in the six treatments. The averages of individually calculated values of all six labeled treatments for each sampling time are reported.

Follow-up experiments to determine whether small additions of glucose had any detrimental effects on larval development and adult emergence rates were undertaken. Only treatments T4, T6 and T7 were studied. Between 250–260 stage 1 larvae were hand-counted into three replicate trays of each treatment. All conditions were identical to those described above. The position of the trays on the heating mats was rotated to avoid any environmental bias. Adult emergence rates were noted daily and adults removed from the trays on a daily basis.

## Results and discussion

It was possible to mark adult male mosquitoes by addition of labeled glucose to the larval diet. The isotope label was sufficiently "fixed" in the adult, that is sufficient label was incorporated into the structural tissues, and was retained at a detectable level throughout the mosquito's adult life (Table 1 and 2). Particularly in treatments T2, T3, T6 and T7 sufficient label was incorporated into the males, suggesting it would be a useful technique for mark-release type experiments and could be used as a discreet and acceptable label for SIT-released insects. The enrichment of the adult mosquitoes was high enough to assume beyond reasonable doubt that the adult mosquitoes were derived from an artificially ^13^C labeled population. In treatments T2, T3, T6 and T7 the average enrichment minus the standard deviation, was at all sampling times greater than 0.0δ‰ vs PDB a value which is rarely observed in terrestrial organic resources in nature and would thus confirm it an artificially-labeled specimen. Inadequate labeling was achieved in treatments T1 and T5 (the lowest enrichments of either method of delivery), although label was detectable in the young adult, the enrichment dropped over time due to metabolic turnover and therefore was insufficient to be used as a distinct marker, as in field conditions similar isotopic signatures maybe observed in mosquitoes which have fed on C_4 _plants. Terrestrial vascular plants differ in the ^13^C/^12^C ratios because of their photosynthetic pathways, C_3_, C_4 _or CAM. C_3 _plants, so-called because the first product in the photosynthetic Calvin cycle pathway is a C_3 _compound, are generally native in temperate climates with lower light intensity, these plants have isotopic values which range between -24‰ and -34‰ vs PDB depending on environmental conditions [24]. Plants with C_4 _Hatch-Slack photosynthetic pathways, that thrive in higher light intensity environments, such as maize, millet etc and have isotopic values in the range of -7 to -18‰ vs PDB with maize about -10 – 13‰ vs PDB. CAM (Crassulacean Acid Metabolism) plants are generally adapted to low water environments, such as succulents or desert plants and have isotopic values in between those of C_3 _and C_4_.

**Table 1:** δ^13^C of whole mosquitoes in treatments T1-T4. Figures in parenthesis are standard deviations. n = number of mosquitoes analyzed; NS = no sample taken.

Age (days)	n	T1 0.5 mg*	T2 2.5 mg	T3 5 mg	T4 Control
Pupae	5	-2.1	45.5	109.2	-20.0
		(2.6)	(8.7)	(6.8)	(0.2)
3	5	-8.7	33.5	76.1	-20.1
		(2.7)	(12.7)	(17.1)	(1.3)
7	5	-14.2	11.8	59.3	-22.6
		(1.7)	(3.5)	(10.8)	(0.3)
14	5	-15.0	13.9	51.3	-22.5
		(0.5)	(5.0)	(15.9)	(0.3)
21	3	-12.5	13.3	21.0	-22.4
		(1.9)	(8.1)	(11.1)	(0.5)
28	3	-12.4			-22.4
		(0.6)	NS	NS	(0.3)

*99 atom % ^13^C dried solid glucose powder per gram of fish food.

**Table 2:** δ^13^C of whole mosquitoes in treatments T5-T7. Figures in parenthesis are standard deviations. n = number of mosquitoes analyzed; NS = no sample taken.

Age (days)	N	T5 0.175 mg*	T6 0.875 mg	T7 1.75 mg
Pupae	5	-5.8	66.4	76.0
		(2.8)	(15.4)	(19.5)
3	5	-11.9	21.5	41.6
		(1.0)	(4.7)	(8.5)
7	5	-16.4	11.7	19.3
		(2.1)	(3.6)	(1.4)
14	5	-16.9	10.5	19.1
		(2.9)	(7.1)	(11.0)
21	3	-14.6	12.8	17.8
		(3.1)	(0.2)	(4.5)
28	3	NS	11.4	NS
			(1.7)	

* 99 atom % ^13^C glucose added to tray as one-shot solution, in addition to regular fish food.

The follow-up experiments in which small amounts of glucose were added to larval trays suggested that there were no significant detrimental effects of addition of glucose on larval development, as adult emergence was not significantly different between the three treatments as determined by ANOVA analysis. The percentage adult emergence from the stage 1 larvae was 44% (2), 51% (4) and 44% (2) for the T4, T6 and T7 treatments respectively where the figures in parenthesis are standard deviations of the 3 replicates.

From a mass rearing perspective, the addition of the label in a liquid form as implemented in treatments T5-T7 would be more appropriate than mixing the solid labeled glucose with the dried food as in treatments T1-T3, as producing a homogenous labeled food source would prove difficult in a large-scale operation. Labeled liquid solutions could easily be made on a large scale, measured out, batched up, stored frozen and added to the larval trays on thawing, ensuring constant level of label addition.

**Figure 1:**
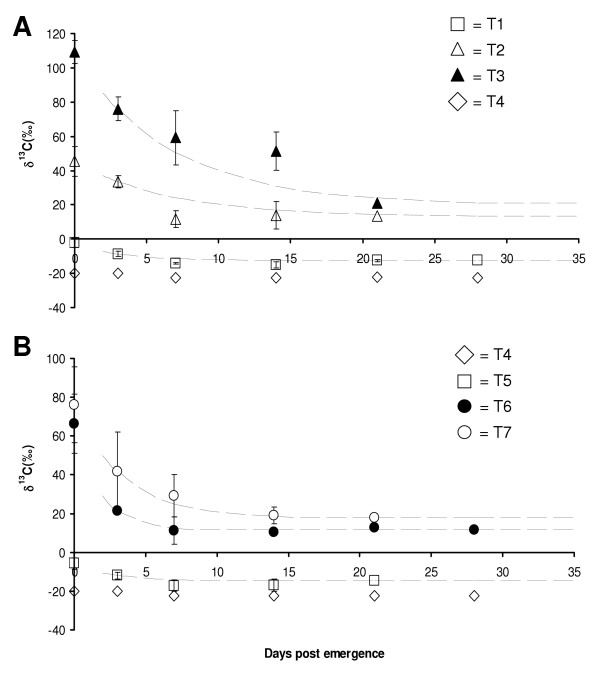
δ^13^C of whole mosquitoes (± standard deviation) against time after emergence (days) for treatments T1-T4 (A) and T4-T7 (B). The dashed lines represent the modeled data.

**Figure 2:**
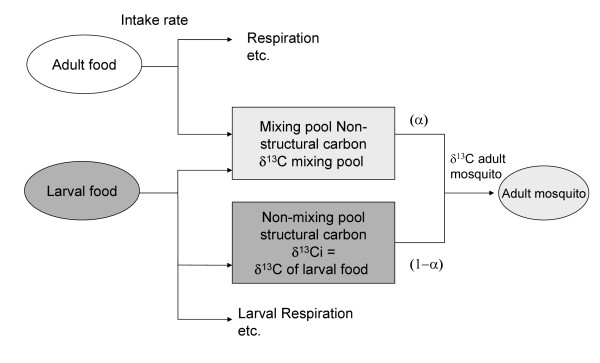
Graphic representation of mosquito ^13^C composition.

It was possible to study the metabolic turnover of the adult male mosquitoes using the isotopic data. Although isotopic enrichments in treatments T3 and T7 decreased exponentially with r^2 ^= 0.92 and 0.84 respectively, this model did not fit the data of the remaining treatments adequately (Figures 1A and 1B). An alternative two-pool model of mosquito isotopic composition was formulated (Figure 2) which consisted of a dynamic pool and a static pool; a similar model was described by O'Brien et al.,[21] and Fischer et al., [22] and applied to nectar feeding in Lepidopterans. The static pool being representative of ^13^C incorporated into the mosquito's structural carbon, i.e. chitin and the dynamic pool representative of the fraction which turns over rapidly i.e. blood sugar, glycogen, etc. Using this model it was possible to calculate an average turnover rate of the dynamic carbon fraction (r), which ranged from 0.15 – 0.57 day^-1 ^across treatments. This model provided a closer fit to the observed data in the labeled treatments overall, with the r^2 ^of the modelled versus actual values shown in Figure 1 and Table 3.

**Table 3:** Fractional turnover rate as determined by the model equation (4) and r^2 ^of modeled plotted against observed values.

Treatment	Fractional turnover rate r day^-1^	Modeled versus observed value r^2^
1	0.34	0.6563
2	0.15	0.6984
3	0.16	0.8011
5	0.40	0.6411
6	0.57	0.9498
7	0.30	0.9665

δ^13^C mosquito at time t = e^-rt^(δ^13^C_f _- δ^13^C_i_) + δ^13^C_f _    (4)

Where δ^13^C_i _is the ^13^C of the pupae, t = time in days, δ^3^C_f _is the δ^13^C of the mosquito at equilibrium in this case the final sampling time. In treatments T3 and T7, equilibrium may not have been reached by the final sampling and explain why an exponential model fitted the data more closely. It is recognized that a longer and more intensive sampling strategy, which accounts for isotopic fractionation at trophic level, would be necessary to validate this model and determine the turnover rate of the dynamic fraction more accurately but this was not within the scope of this study. Chamberlain et al., [25] have used similar dietary isotopic switching techniques and compound specific gas chromatograph mass spectrometry to study carbon turnover in specific carbon fractions in Collembola, and their results suggest that a similar approach could be applied to mosquitoes.

It was also possible to calculate the % C derived from the sugar diet throughout the lifespan of the mosquito independent of labeling treatment using equation 2. On average, after 3 days, 31% of the adult mosquito carbon was derived from the sugar water fed to the adult mosquitoes, whilst after 7–10 days this figure levelled out to around 50% and rose only slightly over the following two weeks to 53% suggesting that approximately 50–53% of the male mosquito body carbon is turning over and is metabolically active and that approximately 47–50% of the body carbon is structural and does not turnover within the lifespan of the male mosquito under laboratory conditions. These values are consistent with carbon supply data from similar isotopic studies of Lepidoptera which showed that after one week 50–60% egg producing resource was derived from nectar sugars [21]. The reported experiments suggest that approximately 49–50% of the mosquito carbon is structural and that approximately 50–51% metabolically active. Using these data and equation 3 it was then possible to calculate the proportion of C in the metabolically-active fraction derived from the adult fed sugar over time. This was, averaged over the 6 labeled treatments, 74 % (8), 94% (6), 96% (5) and 96% (11) at three, seven, 14 and 21 days post-emergence, respectively, suggesting that there is significant sugar feeding following emergence and rapid turnover of the non structural pool. The variation in values could be attributed to the size of the adult and their respective demand for energy supplies [26]. These data again corroborate the two-pool model hypothesis.

As the mosquitoes switched to the unlabeled sugar resource, the isotope enrichment of the individual mosquitoes declined according to the formula described by the model in which there was exponential decay followed by flattening of the curve. It was generally possible to distinguish between three and seven-day old mosquitoes based on the isotopic signature alone (based on T tests analysis not shown P =< 0.05), however it was not possible to tell seven and 14-day old mosquitoes apart in all treatments due the error associated with the average values and the proximity to the leveling off point of the curve. In treatments T3 and T7, enrichment of the mosquito dropped to half its original value in 10.34 and 10.69 days respectively, yet in treatments T1, T2, T5 and T6 reduction to half initial enrichment was less than five days; this interval will be determined by the original enrichment of the larval feed and the rate of dilution or uptake of adult feed. This suggests that it will only be possible to distinguish age classes in the initial stages of adult life (1–10 days). In treatments T1 and T5, the errors associated with the average values were considerably lower; this can be explained by the influence of initial enrichment of the larvae and the dilution effect and comparative versus absolute values. This suggests the technique has potential as an age-grading tool for male mosquitoes in natural habitats over a short age range; as the larval breeding habitats and adult feeding resources are likely to have distinctively different isotopic signatures. However, the major limitations of the technique are the natural variability in individual fractional turnover rates and adult feeding habits, the variation in initial enrichment of the larval source and the fact that the model assumes a constant rate of resource uptake. These parameters would require semi-field studies followed by field validation prior to recommendation as an aging tool and it may be useful to include the use of additional isotopes such as nitrogen (^15^N).

Given the scale of SIT mass rearing operations, mark-recapture studies and the logistics involved, the cost of the label for marking purposes would not be prohibitively expensive. Based on the data from treatment T6 cost of labeling would be less than 250 US$ per million mosquitoes labeled. Costs of isotope analysis can be as low as 5 US$ per sample and may come down further given the current developments in laser technology. In addition, sample preparation for analysis is minimal as the whole mosquito is put into the tin cup and analyzed; these samples are then dried and once the tin cups are sealed can easily be transported for analysis.

The methods of labeling described here would also be appropriate for mark-release-recapture type studies for investigating dispersal range and population dynamics [11]. The decline in the isotope marker gives an indication of the age of the insect and may be useful as it allows for sequential multiple releases since mosquitoes from different cohorts to be identified.

## Conclusion

Labeling anopheline mosquitoes with ^13^C in the larval diet is both feasible and affordable and can serve as a useful tool for studying both mosquito populations and the fate of released insects in genetic control programmes. Labeled resource acquisition of male adult mosquitoes showed that labeling with ^13^C can also be a useful tool for studying carbon turnover in mosquitoes. Given these findings it is suggested that such labeling techniques could be extended to specific metabolic fractions of such as glycogen or lipid fractions [25], thus broadening the scope of stable isotope use in mosquito biology.

## Authors' contributions

RHN planned and implemented the experiments and wrote the first draft of the document, LM did the isotope analysis, and BGJK supervised the work and contributed significantly to the final draft of the paper.
